# Immunolocalization of cyclotides in plant cells, tissues and organ supports their role in host defense

**DOI:** 10.1007/s00425-016-2562-y

**Published:** 2016-07-09

**Authors:** Blazej Slazak, Małgorzata Kapusta, Sohaib Malik, Jerzy Bohdanowicz, Elżbieta Kuta, Przemysław Malec, Ulf Göransson

**Affiliations:** 1 W. Szafer Institute of Botany, Polish Academy of Science, 46 Lubicz St, 31-512 Cracow, Poland; 2 Division of Pharmacognosy, Department of Medicinal Chemistry, Uppsala University, Biomedical Center, Box 574, 751 23 Uppsala, Sweden; 3 Department of Plant Cytology and Embryology, Faculty of Biology, University of Gdańsk, 59 Wita Stwosza St, 80-308 Gdańsk, Poland; 4 Department of Plant Cytology and Embryology, Institute of Botany, Jagiellonian University, 9 Gronostajowa St, 30-387 Cracow, Poland; 5 Faculty of Biochemistry, Biophysics and Biotechnology, Jagiellonian University, 7 Gronostajowa St, 30-387 Cracow, Poland

**Keywords:** Cyclotides, Immunohistochemistry, Host defense peptides, *Viola*

## Abstract

**The distribution of cyclotides was visualized in plant cells, tissues and organs using immunohistochemistry. Finding of cyclotides in tissues potentially vulnerable to pathogen attacks supports their role as defense molecules.**

The cyclotide family of plant peptides is characterized by the cyclic cystine knot motif and its diverse biological activities. Given their insecticidal and antimicrobial properties, the role of cyclotides *in planta* is probably associated with host defense. Our current understanding of the cellular compartmentalization of cyclotides in the vacuole is based on indirect studies on transgenic model plants that do not express cyclotides naturally. Matrix-assisted laser desorption ionization (MALDI) imaging has also been used to study the distribution of cyclotides, but the technique’s resolution was insufficient to determine their tissue or cell distribution. To avoid the limitations of these approaches, immunohistochemical visualization methods were used. Antibodies were raised in rabbits using cycloviolacin O2 (cyO2), and their specificity was determined by Western and dot blot experiments. Slides for immunohistochemical analysis were prepared from leaf, petiole and root fragments of *Viola odorata* and *Viola uliginosa*, and specimens were visualized using indirect epifluorescence microscopy. The antibodies against cyclotides were specific against selected bracelet cyclotides with high similarity (cyO2, cyO3, cyO8, cyO13) and suitable for immunohistochemistry. The tissue distribution of the cyclotides visualized in this way is consistent with their proposed role in host defense—relatively large quantities were observed in the leaf and petiole epidermis in both *Viola* species. Cyclotides were also found in vascular tissue in all the assessed plant organs. The vacuole storage of cyclotides was directly shown.

**Electronic supplementary material:**

The online version of this article (doi:10.1007/s00425-016-2562-y) contains supplementary material, which is available to authorized users.

## Introduction

**Fig. 1:**
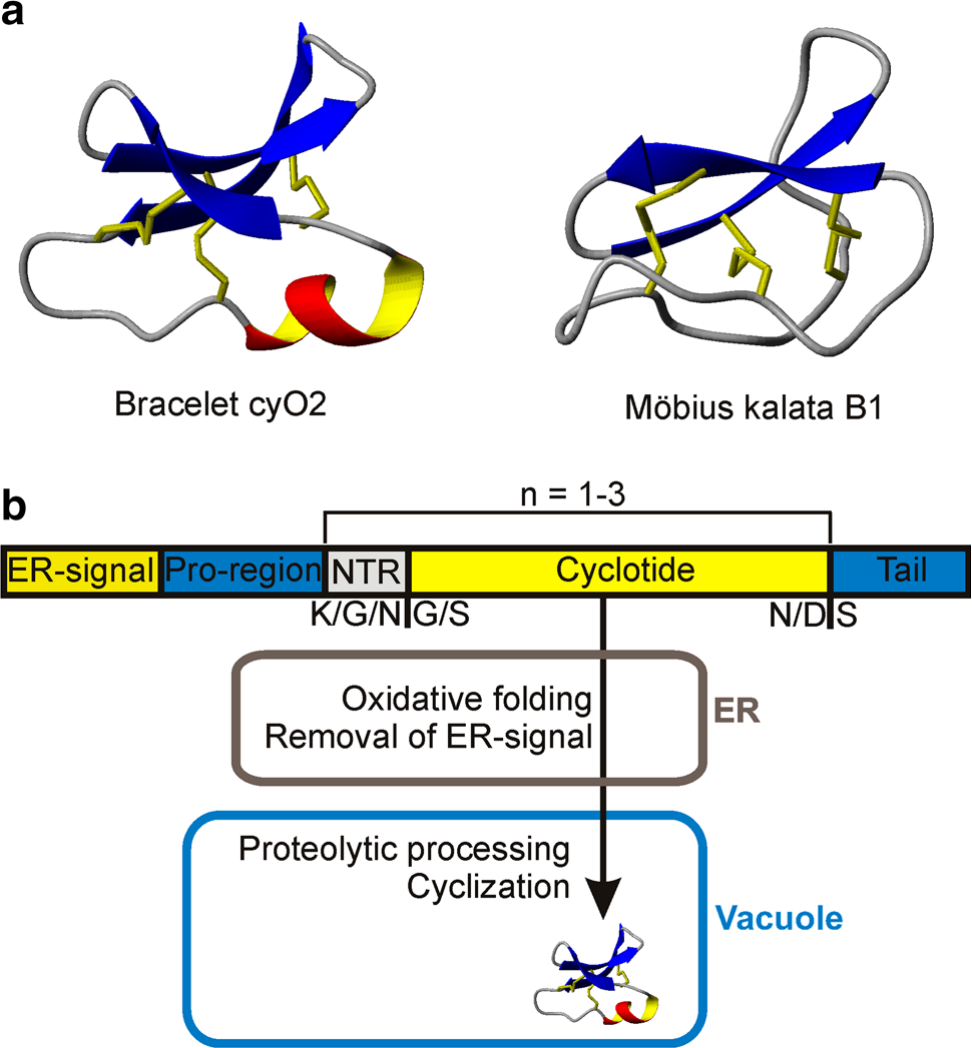
Two types of cyclotides subfamilies and their biosynthesis. **a** 3D structures of bracelet and Möbius cyclotides. **b** Structure of a cyclotide precursor peptide and mature cyclotide formation. Five regions can be distinguished: the ER-signal, pro-region, and N-terminal repeat (NTR), the cyclotide sequence, and a short C-terminal tail. The NTR and cyclotide sequences (the same or different sequences) can be repeated 1–3 times (*n* = 1–3). The mature cyclotide is formed after enzymatic processing in the endoplasmic reticulum (ER) and vacuole (Jennings et al. 2001; Gruber et al. 2007; Gillon et al. 2008; Nguyen et al. 2014)

Cyclotides are head-to-tail cyclic plant peptides consisting of approximately 30 amino acid residues with a characteristic motif known as the cyclic cystine knot (Craik et al. 1999; Göransson and Craik 2003). Two main subfamilies of cyclotides can be distinguished based on their amino acid sequences: the Möbius and the bracelets (Fig. 1a). To date, cyclotides have been found in six families of angiosperms: the Rubiaceae, Cucurbitaceae, Fabaceae, Solanaceae, Poaceae and Violaceae. The Violaceae, is particularly rich in cyclotides, which appear to be expressed in all members of this plant family (Nguyen et al. 2011; Poth et al. 2012; Gerlach et al. 2013; Nguyen et al. 2013; Burman et al. 2014, 2015; Hellinger et al. 2015). The number of individual cyclotides in one species can exceed 150, and the total number of cyclotides in the Violaceae family alone is currently estimated to be over 125 000 (Hellinger et al. 2015). While the details of the biosynthesis and functions of these peptides are gradually being revealed, we still lack fundamental knowledge about their localization and distribution *in planta*.

Cyclotide biosynthesis involves the expression of precursor proteins (Fig. 1a) followed by a complex set of posttranslational processing steps that occur in different cell compartments (Fig. 1b). The precursor peptides are synthesized on ribosomes, the enzymatic removal of the ER-signal peptide and oxidative folding occur in the endoplasmic reticulum (Jennings et al. 2001; Gruber et al. 2007; Craik and Malik 2013), and the final steps—enzymatic cleavage of the NTR region and cyclization—are apparently carried out in the vacuole by enzymes residing there (Conlan et al. 2011; Nguyen et al. 2014; Harris et al. 2015). The presence of an ER signal in the cyclotide precursor proteins (Fig. 1a) indicates that they travel through the secretory pathway, which leads to apoplast or vacuole (Vitale and Denecke 1999). However, there is no direct evidence whether the vacuole is the place of deposition and storage of cyclotides, neither in transgenic model species or cyclotide-producing plants.

Cyclotides have diverse biological activities. They were originally discovered because of their uterotonic activity (Gran 1973; Gran et al. 2008), and were later shown to be cytotoxic and active against HIV (Burman et al. 2014). Their role as plant defense molecules was suggested on the basis of their insecticidal (Jennings et al. 2001) and antimicrobial (Pränting et al. 2010) activity. The tissue and organ distribution of defense compounds can be linked to their natural role. For example, compounds present in the epidermis are likely to protect the plant against small insects and microorganisms such as bacteria and fungi that must cross this first barrier at the onset of infection. Conversely, defensive metabolites occurring in the mesophyll may provide protection against larger herbivores that feed on whole plant organs (Nuringtyas et al. 2012).

Peptide extraction and analysis experiments have shown that cyclotide expression differs between plant organs (Göransson et al. 2003; Trabi and Craik 2004), and the regulation of cyclotide expression seems to be related to the activity of plant growth regulators (Slazak et al. 2015a). However, little is known about the distribution and occurrence of cyclotides within organs and tissues; the only published study on this topic used MALDI imaging to study the distribution of these peptides in the leaves of *Petunia* (Solanaceae) (Poth et al. 2012). Both MALDI imaging and biotechnological alternatives such as Green Fluorescent Protein (GFP) labeling of cyclotide precursors (Conlan et al. 2011) have limitations that reduce their usefulness as tools for studying the cellular compartmentalization and tissue distribution of cyclotides. The resolution of MALDI imaging is too low to distinguish different tissues in cross section slides the maximum resolution achievable with this technique is 5 µm, which is not sufficient to identify individual cell compartments (Cillero-Pastor and Heeren 2014; Aichler and Walch 2015). GFP labeling introduces a large non-native group into the peptide, and requires a suitable system for transformation: currently this has only been attempted in one model plant that does not naturally express cyclotides (Conlan et al. 2011). It would thus be desirable to develop alternative methods based on immunohistochemical techniques.

In the current study, we aim to show that cyclotides are stored in the vacuole and demonstrate cyclotides distribution in plant tissues and organs using immunohistochemistry. To this end, antibodies were raised against the bracelet cyclotide cycloviolacin O2 (cyO2), which is widely distributed in species of the Violaceae (Burman et al. 2015).

## Materials and methods

### Plant material

Specimens of *Viola uliginosa* Besser were obtained as previously described (Slazak et al. 2015b). *Viola odorata* L. was collected by Prof. Elżbieta Kuta during the summer of 2014 and then maintained under laboratory conditions with a controlled temperature and photoperiod (Gdańsk University). *Arabidopsis thaliana* L. Heynh. (ecotype Columbia) and *Nicotiana benthamiana* (Domin) were cultivated in the laboratory with a controlled temperature and photoperiod.

### Cyclotide extraction

Cycloviolacin O2 (cyO2) was extracted from dried plant material of *V. odorata* using a previously reported protocol (Herrmann et al. 2008). Briefly, after three rounds of extraction with fresh 60 % aq. methanol, the extract was partitioned with dichloromethane. Then, to separate cyO2, positively charged molecules were captured from the (three times diluted) aqueous layer of the partitioned extract using solid phase strong cation-exchange extraction.

The plant extracts were fractionated using a Waters 600 HPLC system (Waters Corporation, MA, USA) fitted with a Phenomenex Jupiter C18 column (250 × 21.2 mm i.d., 10 µm, 300Å). Elution was performed using a linear gradient from 10 % acetonitrile (ACN) containing 0.05 % trifluoroacetic acid (TFA) (buffer A) to 60 % ACN containing 0.05 % TFA (buffer B) over 45 min, with a flow rate of 15 ml/min. Fractions were analyzed by ESI–MS (Finnigan LCQ ion trap, Thermo Electron Co., Waltham, MA, USA) in positive ion mode.

Cyclotide-containing fractions were subjected to a second purification step using an ÄKTA basic HPLC system (Amersham Biosciences, Uppsala, Sweden) fitted with a Phenomenex Jupiter C18 column (250 × 10 mm i.d., 5 µm, 300Å). Elution was performed using a linear gradient from 40 to 70 % of buffer B at a flow rate of 4 ml/min. Fractions were analyzed by ESI–MS and the pure fractions were freeze-dried. The purity of the isolated peptide was determined using a nanoAcquity Ultra Performance LC system (Waters Corporation, MA, USA).

CyO3, cyO8, and cyO8 were obtained from *V. uliginosa* using the method described previously (Slazak et al. 2015a).

### Raising of antibodies

Polyclonal anti-cyclotide antibodies were raised in rabbit using standard procedures (Capra Science Antibodies AB, Ängelholm, Sweden). Immunization was performed with a mixture of free cyO2 and cyO2-conjugated keyhole limpet hemocyanin (KLH). Two rabbits were immunized with approx. 500 μg of this mixture per animal over the complete 12-week immunization period, which featured an initial immunization on week 0 followed by immunization boosts on weeks 2, 4, 7, and 10. The rabbits were bled on weeks 6, 9, and 12 (10 mL antiserum/rabbit). Antisera from the two rabbits were then pooled and purified using an affinity column to which 1–2 mg of the antigen was coupled. The eluted antibodies were dissolved in water (0.15 mg/ml) and stored at −20 °C.

### Dot blot and Western blot experiments to confirm the antibodies’ specificity

Petioles and leaves of *V. odorata,* *V. uliginosa, A. thaliana* and *N. benthamiana* were snap frozen in liquid nitrogen immediately after collection and ground to a powder in a mortar. The resulting material was then homogenized in an ice-cold lysate buffer containing 50 µM Tris HCl (Bio-Rad, Hercules, CA, USA), 150 mM NaCl (Sigma-Aldrich, St. Louis, MO), 1 % v/v Triton X (Bio-Rad), 5 mM Dithiothreitol (DTT, Sigma-Aldrich), 5 mM phenylmethylsulfonyl fluoride (PMSF, Sigma-Aldrich) and 1 mM ethylenediaminetetraacetic acid (EDTA, Sigma-Aldrich), pH 7.6. The homogenate was centrifuged at 12,000 rpm for 10 min at 4 °C, and the supernatant was collected. The protein concentration in the supernatant was determined using a Lowry protein assay with acetone protein precipitation according to a procedure described by Olson and Markwell (2001). Lysates from *V. uliginosa* and *V. odorata* each containing 25 µg of total protein and a solution of pure cyO2 containing 2 µg of the peptide were mixed in a ratio of 4:1 with Laemmli Sample Buffer (5×), boiled for 10 min to achieve denaturation, loaded onto a 15 % acrylamide (Bio-Rad) casted gel (15 well, 0.75 mm thickness) along with 3 µl of PageRuler™ prestained protein ladder (Thermo Fisher Scientific, Waltham, MA, USA), and separated by electrophoresis (45 min, 200 V). Additionally, as negative controls, *A. thaliana*, *N. benthamiana* and *V. uliginosa* lysates (20 µg protein each) were separated by electrophoresis along with pure cyO2 (1 µg) using a 16 % acrylamide casted gel (1 h, 200 V). The separated proteins were then transferred onto a nitrocellulose membrane (Whatman Protran BA85, Sigma-Aldrich). Blots prepared with the same methods were stained for whole protein with Ponceau S (Sigma-Aldrich). Membranes for dot blot experiments were prepared by blotting 2 µl of purified bracelet (cyO2, cyO3, cyO8 and cyO13) and Möbius (kB1 and kB2) cyclotide solutions of decreasing concentrations in water (200, 100, 50, 25 and 12.5 pmol of peptides per dot). Furthermore, 2 µg of ovalbumin (Sigma-Aldrich) and bovine serum albumin (BSA, Sigma-Aldrich) were dot blotted for probing. The membranes (from both dot and Western blots) were then blocked with 5 % non-fat dry milk in Tris-buffered saline containing 0.05 % Tween 20 (TBS-T) for 1 h at room temperature before being probed with a 1:1000 or 1:500 dilution in TBS-T (for dot blot and Western blot membranes, respectively) of the rabbit anti-cyO2 polyclonal antibody (stock solution at concentration of 0.15 mg/ml) for 3 h at room temperature. After three washes with TBS-T, the membranes were incubated with alkaline phosphatase (ALP)-conjugated goat anti-Rabbit IgG (H&L) (Agrisera AB, Vännäs, SE) (1:4000 dilution of the purchased stock solution in TBS-T) for 1 h at room temperature. After another three washes, the membrane was incubated for 30 min at room temperature with SIGMA FAST™ BCIP/NBT (Sigma-Aldrich) substrate for development.

### Fixation and embedment

Sections/parts of *V. odorata*, *V. uliginosa* (leaf blades, petioles, roots), *A. thaliana* and *N. benthamiana* (leaf blades, petioles) were fixed in 4 % formaldehyde (freshly prepared from paraformaldehyde) and 0.25 % glutaraldehyde in microtubule stabilizing buffer (MSB) for 4 h at room temperature and then at 4 °C overnight, immediately after cutting. The MSB consisted of 50 mM PIPES (piperazine-*N*,*N*′-bis[2-ethanesulfonic acid]), 10 mM EGTA (ethylene glycol-bis[β-aminoethyl ether]*N*,*N*,*N*′*,N*′-tetraacetic acid), and 1 mM MgCl_2_, pH 6.8 (Świerczyńska et al. 2013). After fixation and three rinses in MSB, the plant material was dehydrated in a graded ethanol series and infiltrated with Steedman’s wax, i.e., a 9:1 (w/w) mixture of polyethylene glycol 400 distearate and cetyl alcohol (Sigma-Aldrich). After wax polymerization, plant material was sectioned (5 or 10 µm) and stretched on microscope slides coated with Mayer’s egg albumin. The wax was then removed with ethanol. Finally, the slides were rehydrated in an ethanol-PBS series and immersed in 0.05 M NH_4_Cl (15 min at room temperature) to reduce background fluorescence.

### Immunostaining and fluorescence microscopy

Sections were preincubated in PBS with 1 % BSA (Sigma-Aldrich) for 45 min to prevent nonspecific binding, then incubated overnight at 4 °C with a solution of the rabbit anti-cyclotide polyclonal antibody Ab Cyo2 that was prepared by diluting a 0.15 mg/ml stock solution at 1:800 with 1 % BSA in PBS. Sections were subsequently rinsed in PBS and incubated for 4 h in a solution of a goat anti-rabbit secondary antibody conjugated with DyLight™ 549 (AS12 2084, Thermo Fisher Scientific), diluted 1:800 in PBS. In addition, double staining was performed to enable visualization of the cytoplasm, using a rat primary antibody against α-tubulin (Ab6161, Abcam, UK) and a goat anti-rat secondary antibody conjugated with FITC (Ab6840, Abcam). The chromatin of the nuclei was stained with 7 μg/ml 4′,6′-diamidino–2-phenylindole dihydrochloride (DAPI, Sigma-Aldrich) in PBS. In negative control experiments, the primary, secondary or both antibodies were omitted. Finally, the sections were cover–slipped using Mowiol medium and viewed with a fully automated upright fluorescent microscope (Leica DM6000 B) equipped with a digital 5 megapixel color microscope camera with an active cooling system (Leica DFC450 C), a selection of lenses (HC PL FLUOTAR 10×/0.30 dry, HCX PL FLUOTAR 40×/0.75 dry and HC PL APO 63×/1,40 oil), and an external light source for fluorescence excitation (Leica EL6000). All of this equipment was controlled using the Leica LAS AF software suite. To verify that staining occurred specifically, a set of control experiments was performed (the procedure without the primary, secondary or both antibodies for the *Viola* species, and a complete procedure applied to *A. thaliana* and *N. benthamiana*). All specimens were viewed/photographed with the following exposure parameters: 40× magnification, RHO cube: 730 ms, gain 1,9 and FIM 55 % (patented Fluorescence Intensity Management—Leica FIM) and DAPI cube: 780 ms, gain 1 and FIM 30 %. The photos were acquired as *Z* stacks (depth throughout the whole specimen—5 µm or 10 µm) and deconvolved using 10 iterations of a 3D non-blind algorithm (Autoquant™) to maximize spatial resolution unless otherwise noted. The images shown in the following sections are maximum projections of the acquired *Z* stacks or 3D projections.

### Histological staining

Sections of the leaves, petioles, and peduncles of *V. uliginosa* and *V. odorata* were fixed in a mixture of 96 % ethanol and glacial acetic acid (3:1; v/v) for 48 h then stored in 70 % ethanol. Handmade transverse sections of organs were double-stained (carmine-iodine green) with 0.4 % alum carmine (Grenacher) for 10 min, rinsed with distilled water until all the free carmine was removed, and then stained with iodine green for 3–4 s (Lillie 1977). The green stain was removed by washing several times in distilled water and the stained sections were mounted in glycerol. This caused the epidermis, parenchyma, and phloem elements to be stained red, while the sclerenchyma and xylem vessels were stained green.

## Results

### Antibodies bind to bracelet type cyclotides with high specificity and efficiency

**Fig. 2:**
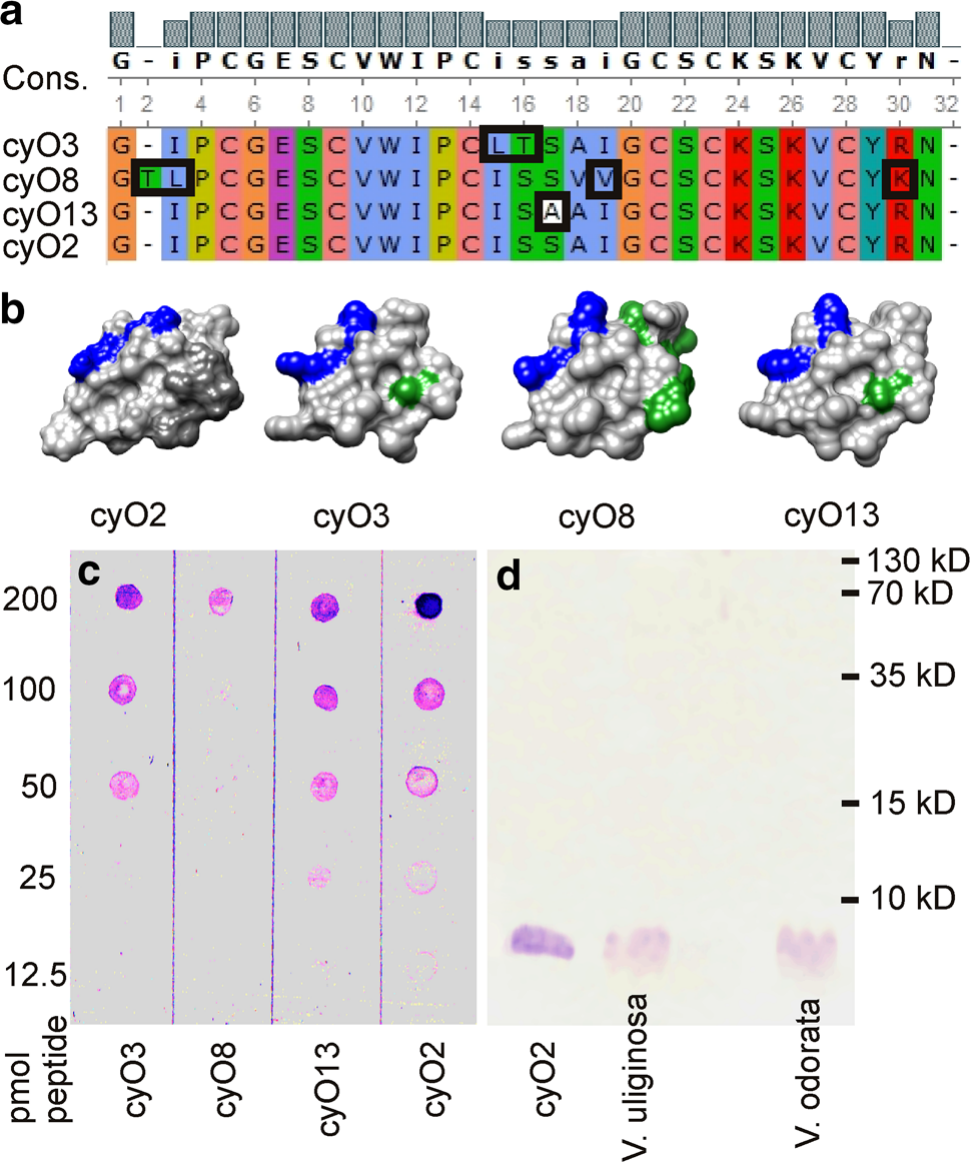
Specificity and affinity of the antibodies raised against cyO2. **a** Clustal X sequence alignment (Jalview coloring scheme) of cyO2, cyO3, cyO8, cyO13 cyclotides used in dot blot experiments. Singular amino acid sequence differences between cyO2 and other cyclotides are highlighted. **b** 3D models of the bracelet cyclotides used in the *dot blot* experiment (cyO2, cyO3, cyO8, cyO13), showing their structural differences. Lysines and arginines are shown in *blue*; residues in loop 3 that differ from their counterparts in cyO2 are shown in *grey* and *green*. **c** Results of *dot blot* experiments. **d** Results of a Western blot experiment showing the specificity of the antibodies—they do not bind to any other proteins from the *V. uliginosa* and *V. odorata* whole protein extracts

Polyclonal antibodies were raised in rabbits using a mixture of cyO2 and cyO2 conjugated to the adjuvant KLH. Antibodies purified using an affinity column containing immobilized cyO2 were then assayed in a dot blot experiment using cyO2, cyO3, cyO8, and cyO13 (Fig. 2a, b).

Antibodies bound with high efficiency to the cycloviolacin cyclotides in general, and cyO2 in particular. With this setup, using a primary antibody concentration as low as 1:1000, it was possible to detect cyO2 at levels as low as 12.5 pmol, which is comparable to the sensitivity of mass spectrometry (Fig. 2c). The antibody’s binding to the bracelet cyclotide cyO13 was 2 times weaker than that to cyO2, while its binding to cyO3 was 4 times weaker than that to cyO2; these compounds could be detected at levels of 25 and 50 pmol, respectively (Fig. 2c). These peptides differ from cyO2 by one and two amino acids, respectively, with the differences being located in loop 3 in both cases (Fig. 2a). For cyO8, which differs by four amino acids from cyO2 (located in loops 3 and 6), binding was detected only for the highest tested amount of peptide (200 pmol) (Fig. 2c). All the sequence differences between cyO2 and the other cyclotides mentioned above relate to residues located on the same side of the molecular surface, as demonstrated by the surface representations shown in Fig. 2b, which suggests that this is the region of the surface that is recognized by the antibodies. No binding was detected for dot blotted ovalbumin and BSA (Supplement 1. a).

The Western blot showed antibodies specificity—no additional bands could be detected neither for *V. uliginosa* and *V. odorata* (Fig. 2d) nor for *A. thaliana* and N. *benthamiana* (Supplement 1. b, c) whole protein extracts.

**Fig. 3:**
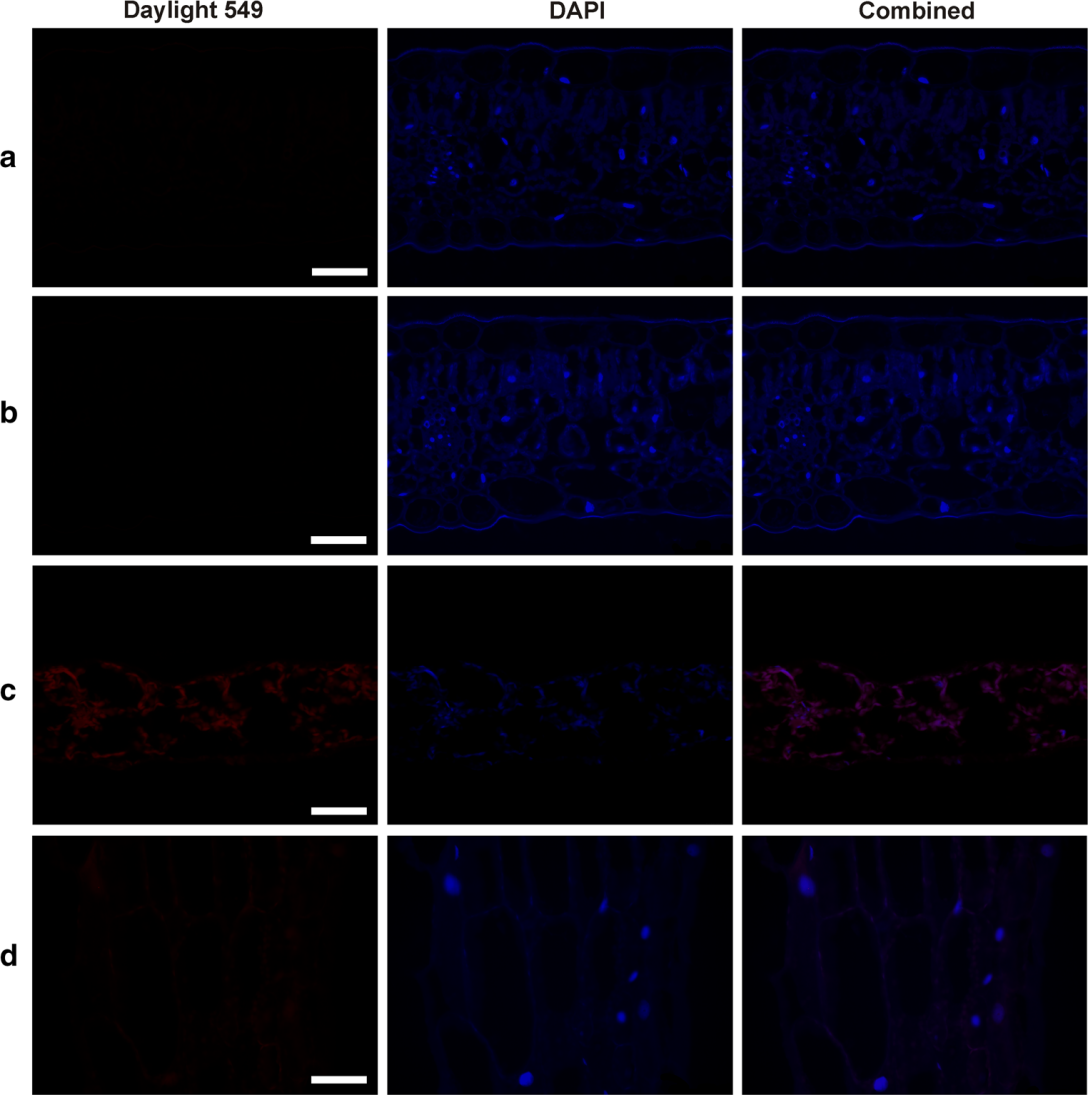
Negative immunostaining controls. **a** and **b** *V. odorata* leaf cross sections, staining with the 2nd but not the 1st antibody (**a**) and with the 1st but not the 2nd antibody (**b**). **c** and **d** *A. thaliana* (**c**) and *N. benthamiana* (**d**) leaf cross sections after the complete immunostaining procedure; only weak autofluorescence due to the chloroplasts is visible in the Daylight 549 channel; *Bar* 100 µm

To further confirm the specificity of the antibodies (both primary and secondary), a series of negative control experiments were performed by staining histological sections of *V. odorata* and *V. uliginosa* without either the primary or the secondary antibody. Additionally, leaf and stem sections of *A. thaliana* and *N.* *benthamiana*, which do not produce cyclotides (Gillon et al. 2008; Conlan et al. 2011), were stained using the same procedures. No specific fluorescence (Daylight 549 channel, red) was detected in any of the resulting slides (Fig. 3a–d), confirming the antibodies’ specificity. The weak red fluorescence seen in the *A. thaliana* and *N.* *benthamiana* slides is due to chloroplast autofluorescence (Fig. 3d, c).

### Immunohistochemistry reveals the distribution of cyclotides in organs, tissues and cells, supporting their proposed role in host defense

**Fig. 4:**
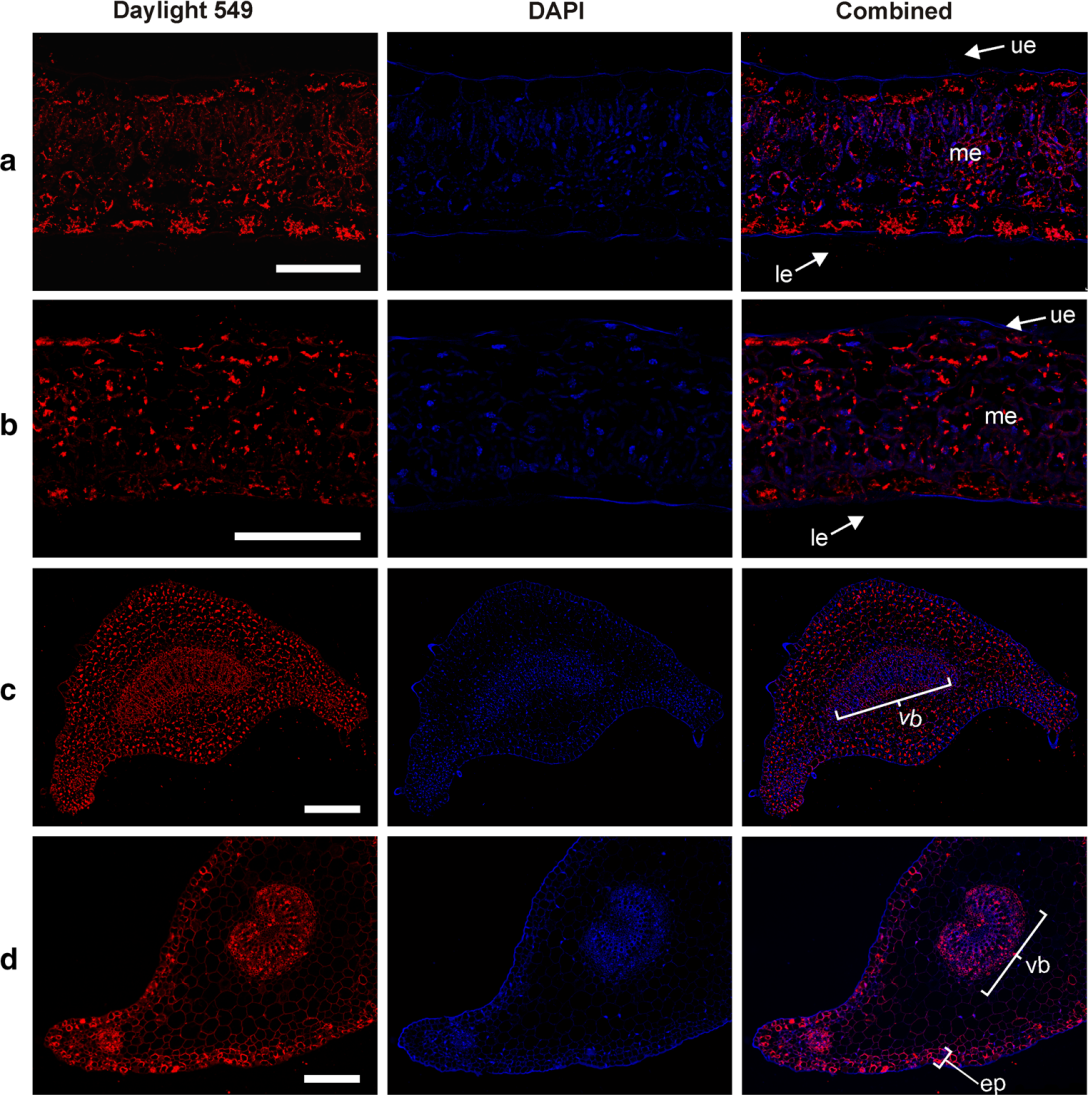
Cyclotide distribution in *V. uliginosa* and *V. odorata* leaves and petioles; the locations of cyclotides are revealed by red fluorescence. **a** and **b** *V. odorata* and *V. uliginosa* leaf cross sections, respectively. In both species, cyclotides are visible in the abaxial (*lower*; le) and adaxial (*upper*; ue) epidermis, and the mesophyll (me). **c** and **d** *V. odorata* and *V. uliginosa* petiole cross sections, respectively. In *V. odorata* (**c**), cyclotides are distributed evenly in the tissues, including the vascular bundle (vb). In *V. uliginosa* (**d**), cyclotides are distributed mainly in the epidermis, subepidermal region (ep), and vascular bundle (vb). *Bar* 100 µm (**a** and** b**) and 200 µm (**c** and **d**)

**Fig. 5:**
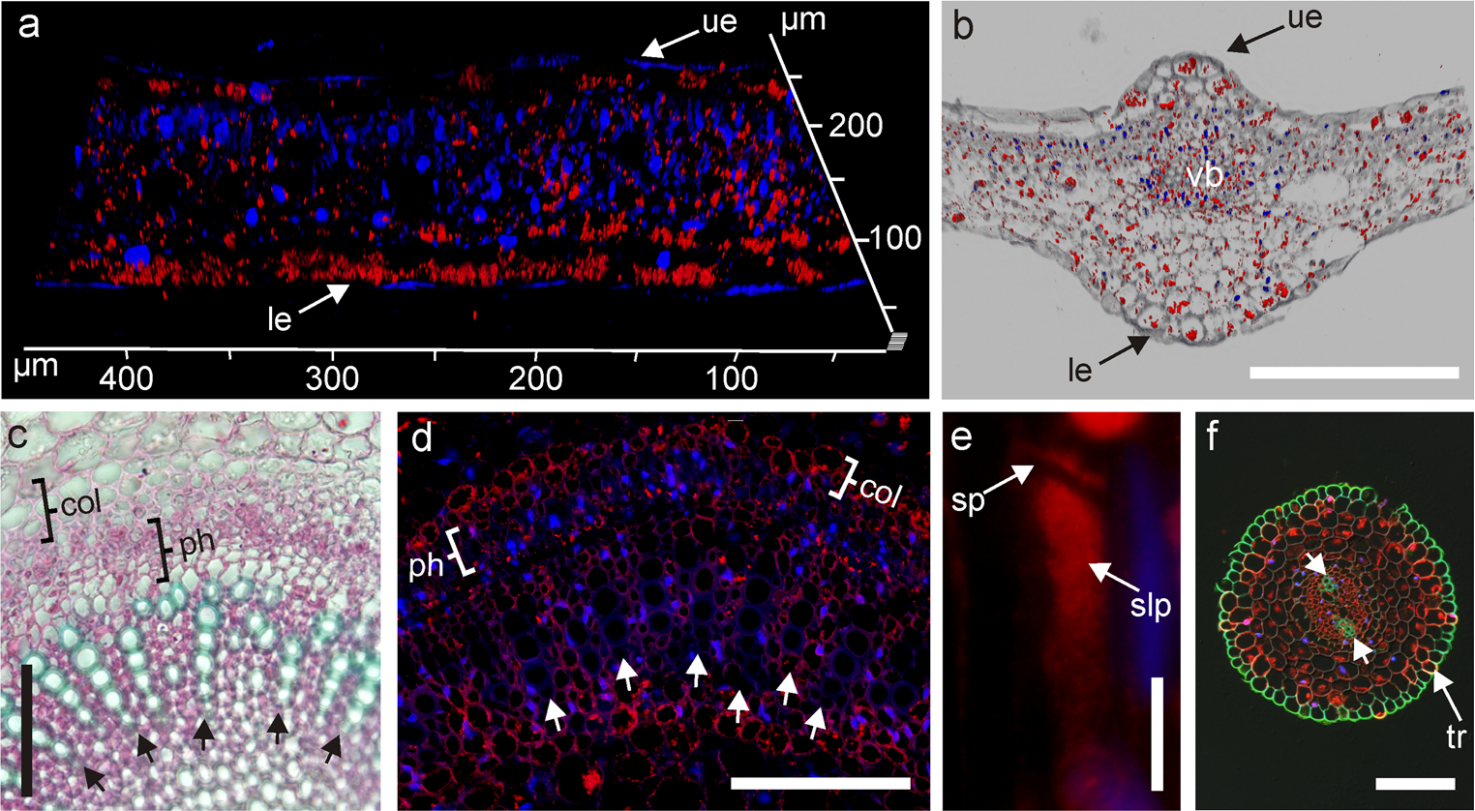
Cyclotide distribution in leaf, petiole and root revealed by red fluorescence. **a** 3-D image from a *z* stack projection of *V. odorata* leaf sections showing the localization of cyclotides in the leaf epidermis and mesophyll; note the large amount of cyclotides in the abaxial (lower; le) epidermis (Daylight 549 and DAPI channels combined, 3D projection of *Z* stack). **b** cyclotide distribution in *V. uliginosa* leaf cross-sections. Cyclotides are present in the abaxial (*lower*; le) and adaxial (*upper*; ue) epidermis, and the vascular bundle tissue (vb). Daylight 549, DAPI and transmission light channels combined (3D projection of *Z* stack). **c** transverse hand-cut sections of the *V. odorata* petiole vascular bundle showing the distribution of different tissues (xylem, phloem and collenchyma). The dead xylem vessels with their lignified cell walls are stained *green* (marked with *arrows*), while the xylem parenchyma (the tissue between xylem vessels), phloem tissue (ph), and collenchyma surrounding the vascular bundle (col) are stained *red*. **d** magnified petiole vascular bundle of *V. odorata* (Daylight 549 and DAPI channels combined) showing cyclotides (red fluorescence) in the xylem parenchyma (between *arrows*), phloem (ph) and collenchyma (col); no cyclotides were found in the xylem vessels (marked with *arrows*). **e** longitudinal section of a sieve element (Daylight 549 and DAPI channels combined) with cyclotides visible in the sieve plates (sp) and slime plug (slp). **f** Cross section of the *V. odorata* root based on the combined Daylight 549 and DAPI channels together with the green channel (wood autofluorescence at 470 nm) to visualize diarchic xylem (*arrows*) and rhizodermis with trichoblasts (tr). In all images, the bars correspond to a length of 100 µm

**Fig. 6:**
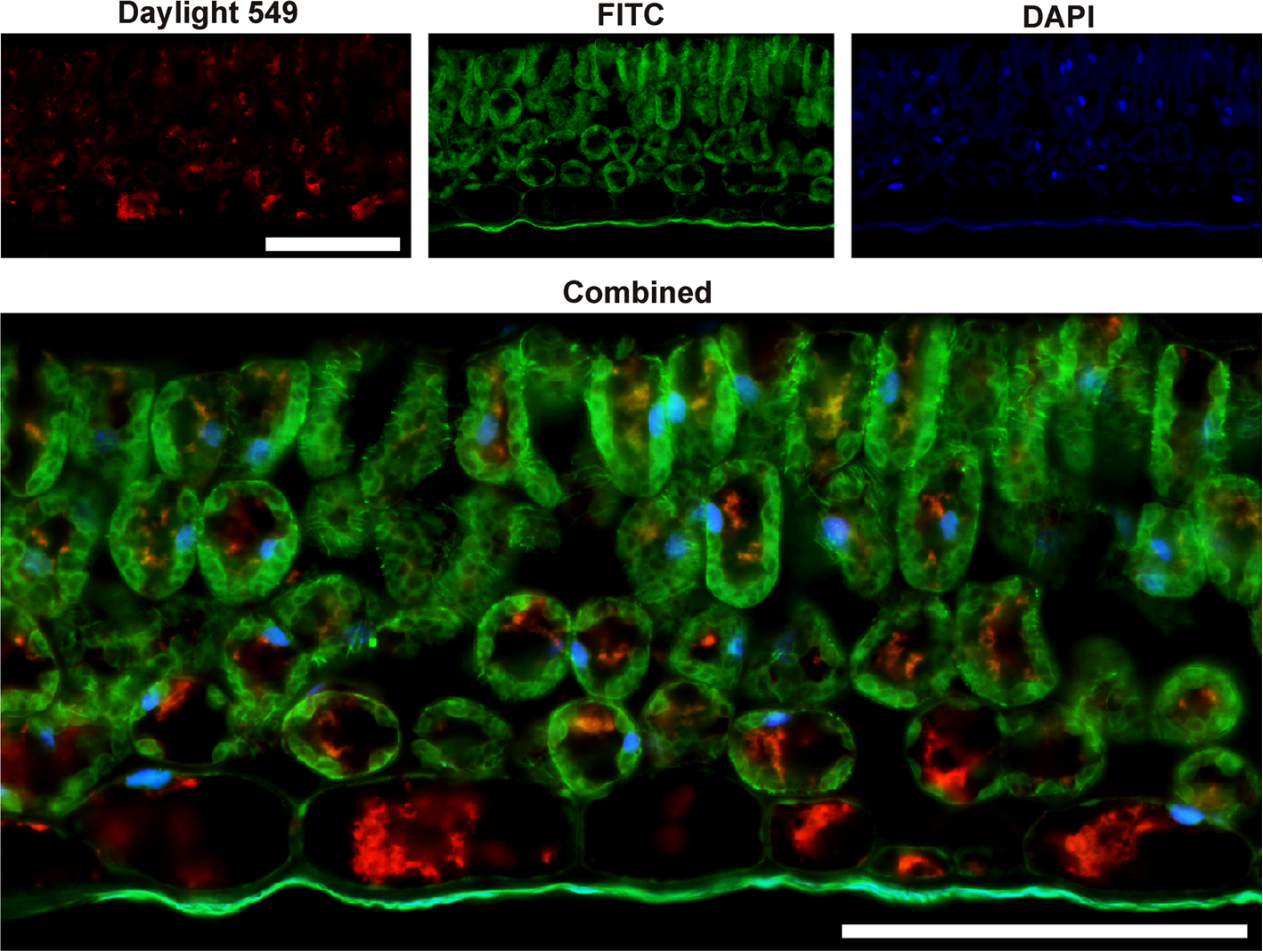
Double immunostaining to visualize the cyclotides’ cellular compartmentalization in *V. odorata* leaves. Cyclotides are indicated by *red* fluorescence (Daylight 549); microtubule distribution in cytoplasm by *green* fluorescence (FITC), and nuclei by *blue* fluorescence (DAPI). *Bar* 100 µm

Cyclotides were detected in all assessed organs—leaves, petioles and roots—in both *V. odorata* and *V. uliginosa* (Figs. 4, 5, 6). Moreover, they were found in tissues vulnerable to pathogen attacks. Large cyclotide concentrations were detected in the epidermis of the leaves of *V. odorata*, especially in the abaxial (lower) epidermis. In addition, cyclotides were detected in the leaf mesophyll, mainly in the spongy parenchyma (Fig. 4a). 3-D *z* stack projections also clearly revealed the patterns of cyclotide distribution in the leaf epidermis and mesophyll of *V. odorata* (Fig. 5a), which differs slightly from that for *V. uliginosa* in that the cyclotides are more evenly distributed throughout the whole organ (Fig. 4b).

Large amounts of cyclotides were found in the petiole tissues of *V. odorata* (epidermis and parenchyma vascular bundle region), as shown in Fig. 4c. In *V. uliginosa* petioles, the content of cyclotides was the highest in the epidermis and subepidermal cells, and also in the vascular bundle region (Fig. 4d). In the roots, cyclotides were present in the cortex parenchyma and trichoblasts but not in the rhizodermis (Fig. 5f). Interestingly, in both species and in all the organs studied (leaf, petiole, root), cyclotides were observed in the vascular bundles (Figs. 4c–d, 5b, d, f). To identify tissues and their co-location in petiole vascular bundles (collenchyma, phloem, xylem vessels and xylem parenchyma), hand-cut double-stained sections (Fig. 5c) were compared to the magnified vascular bundle visible by fluorescent microscopy (Fig. 5d). Cyclotides were detected in the phloem, collenchyma and xylem parenchyma, but not in the xylem vessels. In the phloem, the antibodies bound to proteins of a so-called slime plug which may indicate the presence of cyclotides on sieve plates in sieve tubes (Fig. 5e).

To distinguish between the vacuole and cytoplasm, anti-tubulin antibodies were used to stain the latter. The resulting images clearly show that cyclotides are stored inside the vacuole (Fig. 6).

## Discussion

Very little is known about the distribution of cyclotides in plants that produce them. The main difficulty that has hindered the analysis of their distribution has been a lack of antibodies that bind efficiently to cyclotides, which has prevented the use of immunohistochemical techniques. To the best of our knowledge, only one attempt to raise antibodies against a cyclotide (kB1) has been reported in literature, which was unsuccessful (Conlan et al. 2011; Jennings 2001). Cyclotides are small peptides that may not be immunogenic. One way to overcome such problems is to link a small molecule to a bigger protein. It is most likely that the antibodies obtained by Jennings (2001) were not efficient because kB1 contains no lysine residues via which the cyclotide could be bound to carrier protein. In the current work, a mixture of free antigen (cyO2) and antigen conjugated to KLH (1:1) was used to increase immunization. Conjugation was achieved using the amino groups of the Lys residues in loop 5 of cyO2, which are located on the opposite side of the peptide to the suggested antibody-binding surface. Antibodies prepared in this way were found to be effective, specific to bracelet cyclotides, and suitable for immunohistochemistry. Moreover, they bind to different cyclotide sequences, indicating that they should be useful in a wide range of studies on cyclotide biology, e.g., to track what happens with them in the infected tissues or during embryo development. This incomplete specificity allows visualization of not only cyO2 distribution, but several different cyclotides in Violaceae.

Having obtained a cyclotide-specific antibody, immunohistochemical methods were used to visualize the distribution of these peptides in various *V. odorata* and *V. uliginosa* tissues. Before this there had only been one published study on the distribution of cyclotides in plant organs, which was based on MALDI imaging of Petunia (Solanaceae) leaves (Poth et al. 2012). The findings presented above confirmed the MALDI imaging results in that the studied cyclotides were found to be associated with the leaf vasculature in both works. MALDI imaging offers greater specificity than immunohistochemistry because it can be used to separately visualize the distributions of multiple different compounds on the basis of their molecular masses. However, the imaging resolution achievable with current MALDI methods is limited to approximately 5 µm (Cillero-Pastor and Heeren 2014; Aichler and Walch 2015), which is substantially worse than that achieved using immunohistochemistry in this work. The superior resolution of the immunohistochemical analyses made it possible to study the distributions of cyclotides in individual tissues and cells.

Based on previous reports concerning the behavior of kB1, there were concerns about whether the cyclotides in the plant tissues would remain in their original locations during the ethanol dehydration of the samples in preparation for microscopy (Conlan et al. 2011). In some cases in the current study, cyclotides appeared to form aggregates. This may be caused by the fixation procedure. Cyclotides present at high concentrations in plant cells may precipitate upon treatment with paraformaldehyde or, especially in the case of smaller and more highly dispersed molecules, glutaraldehyde. These aldehydes cause precipitation by cross-linking proteins, primarily via lysine residues (Mannich and Krösche 1912; Richards and Knowles 1968). The cycloviolacin cyclotides examined in this work contain lysine, which may explain why they persist in tissues after ethanol treatment during sample preparation. In contrast, kB1 has no lysines to crosslink, which may explain why its analysis in earlier studies was more challenging. We observed similar bodies of protein/peptide material in our earlier work on *V. uliginosa*, in which we used whole protein staining with naphthol blue black (Slazak et al. 2015a). In keeping with these suggestions, Colgrave et al. (2010) showed that cyclotides can be detected in tissue samples that have undergone the processing steps necessary for histological analysis, provided that they contain at least one lysine residue. In that study, nematodes were exposed to a mutated derivative of kB1 (lysine added to the sequence) linked to a fluorochrome to investigate the anthelminthic activity of the cyclotides. The characteristic fluorescence of the labeled cyclotide was observed in the nematodes’ gut and cuticles even in samples that had undergone complex processing including dehydration in ethanol (Colgrave et al. 2010).

The ability to raise anti-cyO2 antibodies suitable for use in immunohistochemical analysis will not only be important for studies on cyclotides in plants. For example, a recent study showed that a T20 K mutant of the cyclotide kB1 (i.e., a mutant in which the threonine 20 residue was replaced with lysine) is orally available, has immunosuppressive activity, and can be used in multiple sclerosis treatment (Thell et al. 2016). The presence of a lysine in the active cyclotide suggests that it could be linked to KLH to facilitate the raising of antibodies, and will remain in tissue sections as they are processed for histological analysis. This would make it possible to use immunohistochemistry to study the cyclotide’s systemic distribution after ingestion.

In the current work, the cyclotide distribution in the organs (petioles and leaves) was species dependent. In *V. uliginosa*, cyclotides were more abundant in the epidermal region of the petiole and evenly distributed within the leaves whereas the opposite was observed in *V. odorata*, where the cyclotides were evenly distributed in the petiole but present in high concentrations in the leaf epidermis. These distribution patterns may be species-dependent because the two plants belong to different subsections of the *Viola s*ection (*V. odorata* to subs. *Viola*; *V. uliginosa* to subs. *Rostratae*) (Marcussen et al. 2015), and because their morphology have been shaped by different environmental conditions (Valentine et al. 1968).

The results presented above showed that cyclotides are present in the vascular bundles in all of the examined organs (leaves, petioles, and roots) and almost all of the studied tissues (whole phloem, collenchyma and xylem parenchyma, but not xylem vessels). The presence of cyclotides in living cells that contain a tonoplast is unsurprising, as is their absence in xylem vessels. The site of cyclotide deposition and storage is probably the vacuole (Conlan et al. 2011; Nguyen et al. 2014; Harris et al. 2015), so these peptides will only accumulate in living cells that have vacuoles. Xylem vessels, on the other hand, are dead cells with lignified cell walls, and their role is mainly to transport water and minerals from the roots to the above-ground plant organs (Evert 2006).

The presence of cyclotides in the sieve elements will have to be confirmed by collecting phloem sap and analyzing it by mass spectrometry. Although the negative controls performed in this work demonstrate that the raised antibodies are specific for cyclotides, the sheer abundance of proteins in the sieve plates may result in non-specific binding. However, the presence of cyclotides in the phloem sap is not unexpected given the wide variety of different compounds that can be found there, including defense molecules, peptides, macromolecules (proteins and RNA), and sugars (Hoffmann-Benning et al. 2002; Atkins et al. 2011; Hijaz and Killiny 2014). In previous studies using in vitro suspension cultures, cyclotides were found in the extracellular growth medium, suggesting that they can leak from the vacuole to the cell’s exterior (Dörnenburg 2008; Slazak et al. 2015a). A similar mechanism could be responsible for their movement into the phloem sap (i.e. into the sieve tubes) from surrounding tissues. However, Slazak et al. (2015a) attributed the presence of cyclotides in the medium to cell death and disruption rather than active secretion. Moreover, there are very few studies on plants reporting the excretion of substances from the vacuole, and there are only a few hypotheses suggesting mechanisms by which such a process could occur (Echeverría 2000).

It thus seems that cyclotides in the phloem tubes may be remnants from the development and specialization of embryonic or other meristematic tissues. Sieve elements are developed from pluripotent cells through complex differentiation process involving organelle decomposition (Heo et al. 2014). It may be that cyclotides are produced in the early stages of differentiation, when there is still a vacuole, and remain in the phloem sap after the organelles have been broken down. A similar process has been described for some p-proteins (Cronshaw and Esau 1968; Heo et al. 2014).

Another mechanism that could lead to secretion of cyclotides is saturation of the vacuolar sorting receptor. In such case the highly expressed protein is secreted to the extracellular space (Johnson et al. 2007). However, immature, linear cyclotides that bypass the vacuole (where the cyclization takes place) are never found in plants naturally producing those peptides (Gillon et al. 2008).

Because of their biological activities, cyclotides are considered to be defense molecules (Jennings et al. 2001, 2005; Burman et al. 2014). As was shown for other compounds, the organ and tissue distributions of cyclotides may be indicative of their specific roles in host defense; that is to say, a compound’s distribution may indicate which organisms it protects against (Nuringtyas et al. 2012). The high concentrations of cyclotides observed in the epidermis and vascular tissue in this work could thus reflect their antibacterial and antifungal properties described by some authors (Tam et al. 1999; Gran et al. 2008; Pränting et al. 2010; Ovesen et al. 2011; Burman et al. 2014). To infect a plant, these microbial organisms must cross the epidermal barrier; it seems that the cyclotides in the vascular bundle collenchyma and phloem may serve as second line of defense, preventing microorganisms that achieve this crossing from getting into and/or surviving in the phloem sap and then spreading throughout the whole plant. These results also suggest that cyclotides may play a role in defense against sucking animals such as aphids or spider mites. The former imbibe phloem sap while the latter feed by puncturing cells to ingest their contents, usually on the abaxial epidermis of the leaf (Saito 2010). Cyclotides are known to be active against insects (Jennings et al. 2001, 2005), and it looks like the plant concentrates these defense molecules in tissues vulnerable to such attacks. In fact, Gilding et al. (2015) recently showed that the tissue distributions of cyclotides depend on their targets in at least one case: cyclotides produced by the plant *Clitoria ternatea* in its aerial parts, which are exposed to insect herbivores in nature, were found to be active against insect-like membranes whereas those produced in the roots were active against the model soil nematode *Caenorhabditis elegans*.

The results presented herein represent the first successful use of immunohistochemistry to visualize the cellular compartmentalization of cyclotides. All the previous experimental data on this subject came from a study on transgenic *Nicotiana benthamiana* (Conlan et al. 2011), in which conjugates of GFP with precursor peptides (whole sequences or parts) were expressed transiently to study the biosynthetic pathways and final deposition sites of cyclotides in cells. This approach made it possible to track the peptides’ sites of deposition and to characterize the vacuolar targeting region responsible for guiding the construct as it moved through the cell. It was found that the N-terminal region of the cyclotide precursor was sufficient to direct the whole construct to the vacuole (Conlan et al. 2011).

In the future, immunochemical methods will make it possible to track cyclotides in plant tissues under biotic (microbial infections) or abiotic (e.g., due to heavy metals) stress to clarify their role in host defense.

## Conclusion

Antibodies suitable for use in immunohistochemistry were raised against cyclotides, and used to study the distribution of these peptides in tissue sections from two violet species. Cyclotides were found in all tissues except the rhizodermis and xylem vessels. The observed distribution of cyclotides in plant organs supports their proposed role as defense molecules, and their distribution in tissues vulnerable to pathogen attacks suggests that these defenses are localized specifically in sites prone to attack by the organisms against which the individual cyclotides are most active. Double immunostaining enabled direct visualization of the vacuolar storage of cyclotides.

### *Author contribution statement*

BS conceived and designed research; conducted or participated in experiments: dot blot, western blot, cyclotides isolation and purification, immunohistochemistry, microphotography; provided plant material; analyzed data; wrote the manuscript. MK conduced immunohistochemistry and microphotography. SM was responsible for rising of the antibodies. JB supervised the immunohistochemistry experiments. PM supervised western blot and dot blot experiments. EK and UG supervised the research. All authors read and approved the manuscript.

## Electronic supplementary material

Below is the link to the electronic supplementary material.

Supplementary material 1 (PDF 70 kb)
