# Supplementary material for: Immunolocalization of cyclotides in plant cells, tissues and organ supports their role in host defense
Source: Planta. 2016 Jul 9;244(5):1029–40. doi: 10.1007/s00425-016-2562-y (PMC5052299; doi:10.1007/s00425-016-2562-y)

Journal name: PLANTA

Article title: Immunolocalization of cyclotides in plant cells, tissues and organs supports their role in host defense

Authors: Blazej Slazak[1,2], Małgorzata Kapusta[3], Sohaib Malik[2], Jerzy Bohdanowicz[3], Elżbieta Kuta[4], Przemysław Malec[5], Ulf Göransson[2]

Affiliations:

[1]W. Szafer Institute of Botany, Polish Academy of Science, 46 Lubicz St., 31-512 Cracow, Poland

[2]Division of Pharmacognosy, Department of Medicinal Chemistry, Uppsala University, Biomedical Center, Box 574, 751 23 Uppsala, Sweden

[3]Department of Plant Cytology and Embryology, Faculty of Biology, University of Gdańsk, 59 Wita Stwosza St., 80-308 Gdańsk, Poland

[4]Department of Plant Cytology and Embryology, Institute of Botany, Jagiellonian University, 9 Gronostajowa St., 30-387 Cracow, Poland

[5]Faculty of Biochemistry, Biophysics and Biotechnology; Jagiellonian University, 7 Gronostajowa St, 30-387 Cracow, Poland

Corresponding author: Blazej Slazak

[1]W. Szafer Institute of Botany, Polish Academy of Science, 46 Lubicz St., 31-512 Cracow, Poland

phone: +48 660 450 799

e-mail: b.slazak@botany.com

Supplement 1. Additional dot and western blot negative controls confirming the antibody's specificity. **a**, no signal (violet color reaction) can be detected on the membrane with blotted 2 μg of ovalbumin and BSA. **b, c**, western blots stained with Ponceau S for whole blotted protein (**b**) and using the anti-cyO2 primary antibody and a secondary antibody conjugated with ALP (violet color reaction marked with arrowheads)(**c**). Lanes 1, 2, 3 and 4 correspond to *A. thaliana*, *N. benthamiana, V. uliginosa*, and pure cyO2, respectively.

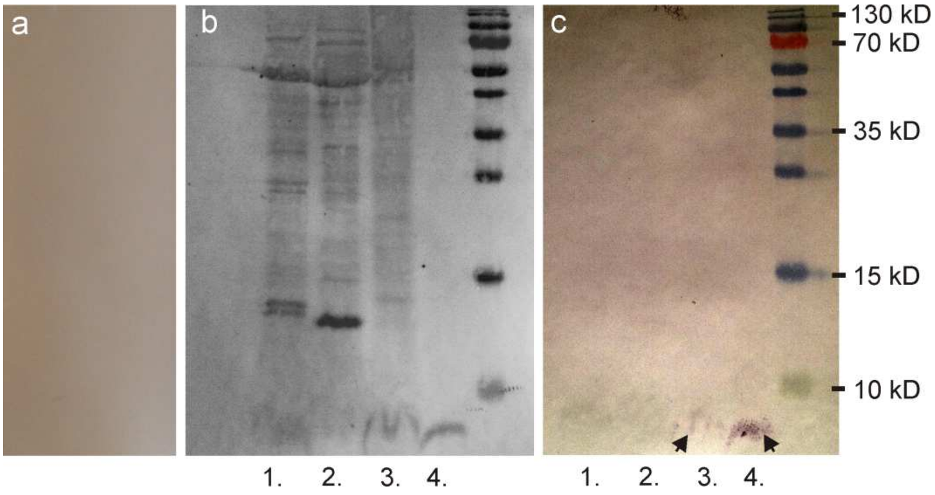

Supplement: Supplementary file 1 — Supplementary material 1 (PDF 70 kb) [file 425_2016_2562_MOESM1_ESM.pdf]
